# Dose approach matter? A meta-analysis of outcomes following transfemoral versus transapical transcatheter aortic valve replacement

**DOI:** 10.1186/s12872-021-02158-4

**Published:** 2021-07-28

**Authors:** Ruikang Guo, Minghui Xie, Wai Yen Yim, Wenconghui Wu, Weiwei Jiang, Yin Wang, Xingjian Hu

**Affiliations:** 1 Department of Cardiovascular Surgery, Union Hospital, Tongji Medical College, Huazhong University of Science and Technology, 1277# Jiefang Avenue, Wuhan, 430022 China; 2 Department of Gastroenterology, Zhongnan Hospital, Wuhan University, Wuhan, China; 3 Department of Gastroenterology, Union Hospital, Tongji Medical College, Huazhong University of Science and Technology, Wuhan, China

**Keywords:** Transfemoral, Transapical, Transcatheter aortic valve replacement, Meta-analysis

## Abstract

**Background:**

Transcatheter aortic valve replacement (TAVR) has gained increasing acceptance for patients with aortic disease. Both transfemoral (TF-TAVR) and transapical (TA-TAVR) approach were widely adopted while their performances are limited to a few studies with controversial results. This meta-analysis aimed to compare the mortality and morbidity of complications between TF- versus TA-TAVR based on the latest data.

**Methods:**

Electronic databases were searched until April 2021. RCTs and observational studies comparing the outcomes between TF-TAVR versus TA-TAVR patients were included. Heterogeneity assumption was assessed by an I^2^ test. The pooled odds ratios(OR) or mean differences with corresponding 95% confidence intervals (CI) were used to evaluate the difference for each end point using a fixed-effect model or random-effect model based on I^2^ test.

**Results:**

The meta-analysis included 1 RCT and 20 observational studies, enrolling 19,520 patients (TF-TAVR, n = 11,986 and TA-TAVR, n = 7,534). Compared with TA-TAVR, TF-TAVR patients showed significantly lower rate of postoperative in-hospital death (OR = 0.67, 95% CI 0.59–0.77, *P* < 0.001) and 1-year death (OR = 0.53, 95% CI 0.41–0.69, *P* < 0.001). Incidence of major bleeding and acute kidney injury were lower and length of hospital stay was shorter, whereas those of permanent pacemaker and major vascular complication were higher in TF-TAVR patients. There were no significant differences between TF-TAVR versus TA-TAVR for stroke and mid-term mortality.

**Conclusions:**

There were fewer early deaths in patients with transfemoral approach, whereas the number of mid-term deaths and stroke was not significantly different between two approaches. TF-TAVR was associated with lower risk of bleeding, acute kidney injury as well as shorter in-hospital stay, but higher incidence of vascular complication and permanent pacemaker implantation.

**Supplementary Information:**

The online version contains supplementary material available at 10.1186/s12872-021-02158-4.

## Background

Transcatheter aortic valve replacement (TAVR) is a recognized alternative to surgical aortic valve replacement (SAVR) with superior in mini-invasiveness and noninferior outcomes of postoperative myocardial infarctions, cerebrovascular events, mid-term mortality and stroke [1]. Trials like PARTNER and CoreValve Pivotal Trial have resulted in a Class I, Level of Evidence: a recommendation for patients with symptomatic severe aortic stenosis (AS) and high surgical mortality risk to undergo TAVR [2, 3]. The Indications of TAVR would be further expanded since some recent RCT trials provide promising interim results in low risk patients [4]. As the exclusive percutaneous approach, transfemoral (TF) access is the most preferred and widely adopted route for TAVR for its safety and less-invasiveness [5]. However, approximately 10–15% of the patients with unsuitable iliofemoral anatomy (iliofemoral arteriopathy, tortuosity, severe calcifications, aortic aneurysm, mural thrombus, previous vascular surgery, or small size) requiring alternative approaches for valve deployment [6]. Differed from the retrograde TF approach, another main access- transapical (TA) TAVR—can be achieved by using anterograde access with left-anterior mini-thoracotomy. TA approach extends the feasibility and broadens indication of TAVR, therefore, it is performed in a reasonable proportion of patients [3]. Nevertheless, TA-TAVR is a more invasive procedure associated with high risk of mortality and morbidity, especially for elder patient with severe comorbidities. Some researchers have suggested that TA-TAVR showed poor outcomes compared with SAVR [7, 8]. While most of the previous studies have assessed the performance of TF and TA approaches separately, comparative studies regarding the safety, efficacy, and efficiency between the two approaches were rarely performed. Thus, we systematically reviewed the latest literature regarding this topic and employed a meta-analytic strategy to determine the short and mid-term mortality as well as incidence of major adverse events between TF- versus TA-TAVR.

## Methods

This meta-analysis was performed in accordance with the PRISMA guidelines statement [9], the MOOSE statement [10] and the Cochrane Handbook Cochrane Handbook recommendations [11]. A systematic literature search was conducted through online databases including PubMed, ClinicalKey, the Web of Science and Google Scholar up till April 2021. For peer- reviewed publications, the language is not limited. The following key words and Medical Subject Headings (MeSH) terms were used: “transcatheter aortic valve replacement (MeSH)”, “transcatheter aortic valve implantation”, “TAVR”, “TAVI”, “transfemoral”, “transapical”, ‘transapical aortic valve implantation’, ‘transfemoral aortic valve implantation’, ‘transapical aortic valve replacement’ and ‘transfemoral aortic valve replacement’. The search string used for PubMed was ‘(((((((((transcatheter aortic valve replacement) OR (transcatheter aortic valve implantation)) OR (TAVR)) OR (TAVI)) AND (transfemoral)) OR (transapical)) OR (transapical aortic valve implantation)) OR (transfemoral aortic valve implantation)) OR (transapical aortic valve replacement)) OR (transfemoral aortic valve replacement)’. References of original articles were reviewed manually and cross-checked. Two investigators (R.G. and M.X.) conducted the search. Two or more studies published from the same database were included if the studies reported outcomes from different follow-up periods or compared different groups.

Studies were included if they fulfilled the following criteria: (1) randomized controlled trials (RCTs) or observational studies published as original articles; (2) compared TF-TAVR versus TA-TAVR; (3) reported at least one of the following events: death (in-hospital, 1-year, and mid-term), stroke, major vascular events, major bleeding, pacemaker implantation, acute kidney injury, reintervention, endocarditis and length of hospital stay; (4) sample size per group of at least 10 patients. Two investigators (R.G. and M.X.) selected the studies for the inclusion, and studies did not meet any of these criteria were excluded. Conflicts between the two investigators were resolved by consensus.

The eligibility and quality of included studies was evaluated independently by two reviewers (Y.W. and X.H.), and a standardized data collection sheet was used for data extraction. Data on investigators, year, journal, design, study period, follow-up duration, procedural approach, sample size, patient characteristics and outcomes were extracted. Disagreements were resolved by consensus. The quality of RCTs and observational studies was appraised by utilizing the components recommended by the Cochrane Collaboration [12], and ROBINS-I (Risk of Bias in Nonrandomized Studies-of Interventions) respectively [13].

The primary outcome of interest was postoperative in hospital death occuring at 1-year as well as 1 to 5 years which is referred to as mid-term mortality. Secondary outcomes included stroke, major vascular events, major bleeding, pacemaker implantation, acute kidney injury.

The pooled odds ratio (OR) or mean difference and corresponding 95% confidence interval (CI) was calculated for dichotomous and continuous outcomes, respectively. Heterogeneity of the studies was assessed using the Higgins I^2^ statistic for each outcome. An I^2^ of 0–25% renders insignificant heterogeneity, 26–50% low heterogeneity, 51–75% moderate heterogeneity and > 75% high heterogeneity [14]. Fixed-effect models of Mantel–Haenszel were used for studies that were homogenous, while Random-effect models of Inverse Variance were used for studies that were heterogenous. Publication bias was assessed visually using a funnel-plot method. Sensitivity analysis was performed by removing studies with the study period finished before 2010. All tests were 2-tailed with a *p* value of < 0.05 considered significant. Analyses were performed using Review Manager Software from the Cochrane Collaboration (Version 5.3, Copenhagen, Denmark).

## Results

**Fig. 1:**
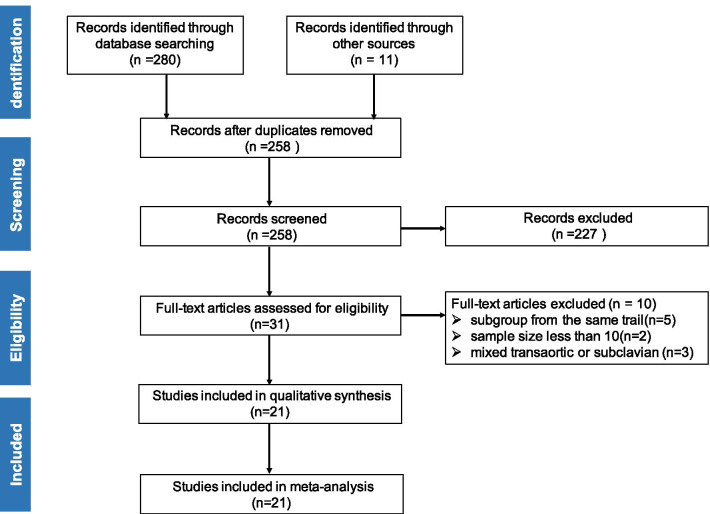
PRISMA study selection flow diagram

Twenty-one studies enrolling 19,520 patients (11,986 undergoing TF-TAVR and 7,534 undergoing TA-TAVR) met the inclusion criteria and were included for the final meta-analysis [6, 15–34]. The search and selection process are shown in Fig. 1. The main characteristics of the included studies are shown in Table 1. Of the 21 studies, 1 was RCT, 9 were prospective observational studies and 11 were retrospective observational studies. The Study quality assessment is summarized in Table 2. The quality of RCT study was high, and among the 20 observational studies, the assessment result of 14 studies was moderate bias, while the remaining 6 studies have serious bias.

**Table 1:** Study characteristics

Lead author	Ayman Elbadawi [34]		Wilko Reents [33]		Mohammed A. Al-Hijji [26]		Takahide Arai [27]		Edward Koifman [28]		Takashi Murashita [21]		Martine Gilard [30]	
Publication year	2020		2019		2019		2016		2016		2016		2016	
Journal	Cardiol Ther		EUR J CARDIO-THORAC		Catheter Cardio Inte		JACC-Cardiovasc Inte		Cardiovasc Revasc Med		Ann Thorac Surg		JACC	
Study design	Retrospective		Retrospective		Retrospective		Prospective		Retrospective		Retrospective		Prospective	
Study period	2011–2014		2009–2016		2012–2016		2011–2014		2007–2014		2008–2015		2010–2012	
Procedure	TF-TAVR	TA-TAVR	TF-TAVR	TA-TAVR	TF-TAVR	TA-TAVR	TF-TAVR	TA-TAVR	TF-TAVR	TA-TAVR	TF-TAVR	TA-TAVR	TF-TAVR	TA-TAVR
Cohort number	2718	2719	619	511	115	115	467	42	516	132	351	216	3064	735
Age, years	78.6 ± 8.5	78.3 ± 8.6	81 (59–95)	81 (67–94)	82.5 ± 7.7	82.8 ± 7.8	83.8 ± 7.1	81.3 ± 7.7	83 ± 8	84 ± 7	79.6 ± 9.7	82.0 ± 7.5	83.2 ± 7.0	81.7 ± 7.5
Male sex	1430(52.6%)	1389(51.1%)	248 (40%)	259 (51%)	60(52.2%)	63(55.3%)	234(50%)	30(71%)	264(51%)	58(44%)	211 (60.1%)	123 (56.9%)	1448(47.3%)	428(58.2%)
STS score,%	N/A	N/A	N/A	N/A	10.0 ± 5.2	10.6 ± 4.7	6.2 ± 3.9	7.1 ± 4.2	8.7 ± 4.5	10.4 ± 4.6	8.8 ± 6.5	9.4 ± 5.4	N/A	N/A
EuroScore,%	N/A	N/A	17 (2–67)	24 (4–79)	N/A	N/A	N/A	N/A	N/A	N/A	N/A	N/A	21.2 ± 14.0	23.9 ± 14.8
Diabetes mellitus	453(16.7%)	503(18.5%)	205 (33%)	177 (35%)	44(38.3%)	46(40.4%)	103(22%)	11(26%)	171(35%)	38(30%)	141 (40.2%)	84 (38.9%)	753(24.7%)	192(26.6%)
Chronic renal failure	1129(41.5%)	1098(40.4%)	345 (56%)	281 (59%)	N/A	N/A	N/A	N/A	N/A	N/A	6 (1.7%)	5 (2.3%)	79(2.6%)	18(2.5%)
COPD	1103(40.6%)	1019(37.5%)	80 (13%)	54 (11%)	77(67%)	70(61.4%)	59(13%)	8(19%)	164(33%)	47(37%)	236 (67.2%)	126 (58.3%)	740(24.3%)	158(21.7%)
Atrial fibrillation	N/A	N/A	157 (25%)	139 (27%)	52(45.2%)	48(42.1%)	132(28%)	18(43%)	212(43%)	52(41%)	N/A	N/A	823(27.6%)	160(22.1%)
Previous stroke	N/A	N/A	76 (12%)	69 (14%)	10(8.7%)	14(12.3%)	13(3%)	2(5%)	60(13%)	29(24%)	83 (23.6%)	71 (32.9%)	286(9.4%)	84(11.7%)
Previous infectious endocarditis	N/A	N/A	N/A	N/A	N/A	N/A	N/A	N/A	N/A	N/A	N/A	N/A	N/A	N/A
Previous valve surgery	N/A	N/A	2 (0.3%)	8 (1.6%)	N/A	N/A	N/A	N/A	N/A	N/A	90 (25.6%)	44 (20.4%)	50(1.6%)	12(1.7%)
Previous myocardial infarction	390(14.3%)	399(14.7)	68 (11%)	78 (15%)	N/A	N/A	15(3%)	2(5%)	N/A	N/A	100 (28.5%)	80 (37.0%)	439(14.4%)	169(23.4%)

Lead author	Vinod H Thourani [24]		Fausto Biancari [22]		Eugene H. Blackstone [25]		Gerhard Schymik [29]		Martyn Thomas [6]		Craig R. Smith[23]		Johan M. Bosmansa[16]	
Publication year	2016		2015		2015		2015		2011		2011		2011	
Journal	Lancet		Am J Cardiol		Circulation		Circ-Cardiovasc Int		Circulation		NEJM		Inter Cardiov Th	
Study design	Prospective		Prospective		Prospective		Prospective		Retrospective		RCT		Retrospective	
Study period	2014.2–2014.9		2010–2012		2007–2012		2008–2012		2007–2009		2007–2009		-2010	
Procedure	TF-TAVR	TA-TAVR	TF-TAVR	TA-TAVR	TF-TAVR	TA-TAVR	TF-TAVR	TA-TAVR	TF-TAVR	TA-TAVR	TF-TAVR	TA-TAVR	TF-TAVR	TA-TAVR
Cohort number	948	126	199	199	501	501	354	354	463	575	492	207	99	88
Age, years	82.1 ± 6.57	80.7 ± 6.69	81.5 ± 6.2	81.2 ± 6.6	218 (44%)	85 ± 6.3	81.7 ± 5.0	81.8 ± 5.9	81.7 ± 6.7	80.7 ± 7.0	84.4 ± 6.7	83.2 ± 6.5	84 ± 5	82 ± 6
Male sex	577(60.9%)	85(67.5%)	111 (55.8%)	104 (52.3%)	283(56%)	272(54%)	164(46.3%)	161(45.4%)	208(44.9%)	254(45.2%)	284 (57.8%)	115 (55.8%)	N/A	N/A
STS score, %	5.3 ± 1.29	5.6 ± 1.28	14.9 ± 11.8	15.0 ± 10.6	N/A	N/A	N/A	N/A	N/A	N/A	11.7 ± 3.3	11.8 ± 3.5	N/A	N/A
EuroScore, %	N/A	N/A	8.1 ± 7.1	8.4 ± 7.3	N/A	N/A	23.5 ± 16.3	23.0 ± 15.6	25.8 ± 14.4	29.1 ± 16.2	29.1 ± 16.1	29.8 ± 15.9	29 ± 15	33 ± 17
Diabetes mellitus	N/A	N/A	52 (26.1%)	50 (25.1%)	184(37%)	185(375)	N/A	N/A	N/A	N/A	N/A	N/A	10(10%)	19(18%)
Chronic renal failure	N/A	N/A	5 (2.5%)	5 (2.5%)	96(195)	92(18)	29(8.2%)	26(7.3%)	118(25.5%)	187(32.5%)	46(9.5%)	16(7.9)	N/A	N/A
COPD	270 (28.5%)	51(40.5%)	44 (22.1%)	50 (25.1%)	221(44%)	214(43%)	46(13%)	47(13.3%)	114(24.6%)	172(29.9%)	211(42.9%)	91(44.0%)	N/A	N/A
Atrial fibrillation	342(36.1%)	43/(34.1%)	N/A	N/A	109(22%)	100(20%)	N/A	N/A	N/A	N/A	106(38.7%)	47(50.5)	N/A	N/A
Previous stroke	81(8.5%)	16(12.7%)	4 (2.0%)	7 (3.5%)	N/A	N/A	N/A	N/A	N/A	N/A	116(25.4%)	66(35.7%)	N/A	N/A
Previous infectious endocarditis	N/A	N/A	N/A	N/A	N/A	N/A	N/A	N/A	N/A	N/A	N/A	N/A	N/A	N/A
Previous valve surgery	51(5.4%)	4(3.2%)	N/A	N/A	N/A	N/A	N/A	N/A	N/A	N/A	N/A	N/A	N/A	N/A
Previous myocardial infarction	133 (14.0%)	39 (31.0%)	54 (27.1%)	53 (26.6%)	136(27%)	140(28%)	46(13%)	47(13.3%)	N/A	N/A	128(26.4%)	67(33.2%)	N/A	N/A

Lead author	See Hooi Ewe [17]		Peter Wenaweser [19]		Rafal Dworakowsk [20]		Josep Rodés-Cabau [31]		Martyn Thomas [15]		Helene Eltchaninoff[18]		Nawwar Al-Attar[32]	
Publication Year	2011		2011		2011		2010		2010		2010		2009	
Journal	Ann Thorac Surg		Am Heart J		Am Heart J		JACC		Circulation		European Heart Journal		Ann Thorac Surg	
Study design	Retrospective		Prospective		Prospective		Retrospective		Retrospective		Retrospective		Prospective	
Study period	N/A		N/A		2007–2009		2005–2009		2007–2009		2009.2–2009.6		2006–2008	
Procedure	TF-TAVR	TA-TAVR	TF-TAVR	TA-TAVR	TF-TAVR	TA-TAVR	TF-TAVR	TA-TAVR	TF-TAVR	TA-TAVR	TF-TAVR	TA-TAVR	TF-TAVR	TA-TAVR
Cohort number	45	59	130	27	67	84	162	177	463	575	161	71	35	15
Age, years	82.2 ± 7.1	79.4 ± 8.3	82.9 ± 5.0	83.9 ± 4.0	83 ± 0.8	82.2 ± 0.8	83 ± 8	80 ± 8	81.7 ± 6.7	80.7 ± 7.0	82.3 ± 7.3	82.1 ± 7.3	83 ± 6	83 ± 10
Male sex	21 (46.7%)	31 (52.5%)	50 (23%)	9 (33%)	43 (51%)	39 (58%)	91 (56.1%)	61(34.5%)	208(44.9%)	254(45.2%)	86(53%)	46(64.7%)	18(51.4%)	9(60%)
STS score, %	8.5 ± 3.8	8.9 ± 3.5	N/A	N/A	N/A	N/A	9.0 ± 5.8	10.5 ± 6.9	N/A	N/A	18.9 ± 12.8	18.4 ± 12.1	15 ± 6	19 ± 9
EuroScore, %	20.1 ± 11.7	22.6 ± 11.9	N/A	N/A	19.4 ± 1.1	23.4 ± 1.5	N/A	N/A	25.8 ± 14.4	29.1 ± 16.2	25.6 ± 11.4	26.8 ± 11.6	26 ± 14	30 ± 12
Diabetes mellitus	13 (28.9%)	16 (27.1%)	27 (20.8%)	8 (29.6%)	18 (26.9%)	17 (20.2%)	37 (22.8%)	42(23.7%)	N/A	N/A	46(28.5%)	18(25.3%)	6(17%)	4(27%)
Chronic renal failure	10 (22.2%)	13 (22%)	N/A	N/A	28 (41.8%)	52 (61.9%)	7 (4.3%)	3(1.7%)	118(25.5%)	187(32.5%)	N/A	N/A	9(26%)	8(53%)
COPD	11 (24.4%)	17 (28.8%)	N/A	N/A	15 (22.4%)	26 (31%)	45 (27.8%)	55(31.1%)	114(24.6%)	172(29.9%)	N/A	N/A	10(29%)	4(27%)
Atrial fibrillation	8 (17.8%)	14 (23.7%)	37 (28.5%)	6 (22.2%)	N/A	N/A	66 (40.7%)	49(27.7%)	N/A	N/A	N/A	N/A	N/A	N/A
Previous stroke	2 (4.4%)	10 (17%)	N/A	N/A	N/A	N/A	27 (16.7%)	50(28.2%)	N/A	N/A	16(9.9%)	6(8.4%)	4(11%)	3(20%)
Previous infectious endocarditis	N/A	N/A	N/A	N/A	N/A	N/A	N/A	N/A	N/A	N/A	N/A	N/A	N/A	N/A
Previous valve surgery	N/A	N/A	N/A	N/A	N/A	N/A	N/A	N/A	N/A	N/A	N/A	N/A	N/A	N/A
Previous myocardial infarction	10 (22.2%)	14 (23.7%)	24 (18.5%)	4 (14.8%)	N/A	N/A	82 (50.6%)	91(51.4%)	N/A	N/A	42(26%)	10(14%)	4(11%)	7(47%)

Data are n (%), or mean ± SD; TF-TAVR = transfemoral transcatheter aortic valve replacement; TA-TAVR = transapical transcatheter aortic valve replacement; COPD = chronic obstructive pulmonary disease; STS = Society of Thoracic Surgeons

**Table 2:** Publication bias analysis

Study (RCT)	Random sequence generation	Allocation concealment	Blinding of participants and personnel	Blinding of outcome assessment	Incomplete outcome data	Selective reporting	Other bias	
Craig R. Smith (2011)	Low	Unclear	Low	Low	Low	Low	Low	
Study (observational)	Bias due to confounding	Bias in selection of participants into the study	Bias in measurement of interventions	Bias due to departures from intended interventions	Bias due to missing data	Bias in measurement of outcomes	Bias in selection of reported result	Overall bias
Ayman Elbadawi (2020)	Serious	Serious	Low	Low	Moderate	Moderate	Low	Moderate
Wilko Reents (2019)	Serious	Low	Low	Low	Moderate	Moderate	Low	Moderate
Mohammed Al-Hijji (2019)	Serious	Serious	Low	Low	Moderate	Serious	Low	Serious
Takahide Arai (2016)	Serious	Low	Low	Low	Moderate	Moderate	Low	Moderate
Edward Koifman (2016)	Serious	Moderate	Low	Low	Moderate	Moderate	Low	Moderate
Takashi Murashita (2016)	Serious	Low	Low	Low	Low	Moderate	Low	Moderate
Martine Gilard (2016)	Serious	Low	Low	Low	Low	Serious	Low	Moderate
Vinod H Thourani (2016)	Serious	Low	Low	Low	Moderate	Serious	Low	Moderate
Fausto Biancari (2015)	Serious	Moderate	Moderate	Low	Moderate	Serious	Low	Serious
Eugene H. Blackstone (2015)	Serious	Low	Low	Low	Moderate	Moderate	Low	Moderate
Gerhard Schymik (2015)	Serious	Low	Low	Low	Serious	Moderate	Low	Moderate
Martyn Thomas (2011)	Serious	Low	Moderate	Moderate	Serious	Moderate	Low	Serious
Johan M. Bosmansa (2011)	Serious	Low	Moderate	Low	Serious	Serious	Low	Serious
See Hooi Ewe (2011)	Serious	Moderate	Low	Low	Low	Serious	Low	Moderate
Peter Wenaweser (2011)	Serious	Low	Moderate	Serious	Moderate	Serious	Low	Serious
Rafal Dworakowski (2011)	Serious	Moderate	Moderate	Low	Serious	Moderate	Low	Moderate
Josep Rodés-Cabau (2010)	Serious	Low	Low	Low	Low	Moderate	Low	Moderate
Martyn Thomas (2010)	Serious	Low	Low	Low	Serious	Moderate	Low	Moderate
Helene Eltchaninoff (2010)	Serious	Low	Low	Low	Serious	Moderate	Low	Moderate
Nawwar Al-Attar (2009)	Serious	Serious	Moderate	Low	Moderate	Moderate	Low	Serious

Publication bias and heterogeneity for each outcome are listed in Table 3.

**Table 3:** Test of heterogeneity and publication bias for each outcome

Outcomes	Chi-square	df	P value	I square (%)	Heterogeneity	Publication bias
In-hospital mortality	34.60	19	0.02	45	Low	None
1-year mortality	1.84	4	0.77	0	Insignificant	None
Mid-term mortality	17.08	3	0.0007	82	High	None
Major vascular complication	66.67	12	< 0.00001	82	High	None
Pacemaker implantation	27.92	16	0.03	43	Low	None
Major bleeding	50.97	10	< 0.00001	80	High	None
Acute kidney injury	72.75	13	< 0.00001	82	High	None
Length of hospital stay	22.57	7	0.002	69	Moderate	None
Stroke	14.01	11	0.23	22	Low	None

### Mortality

**Fig. 2:**
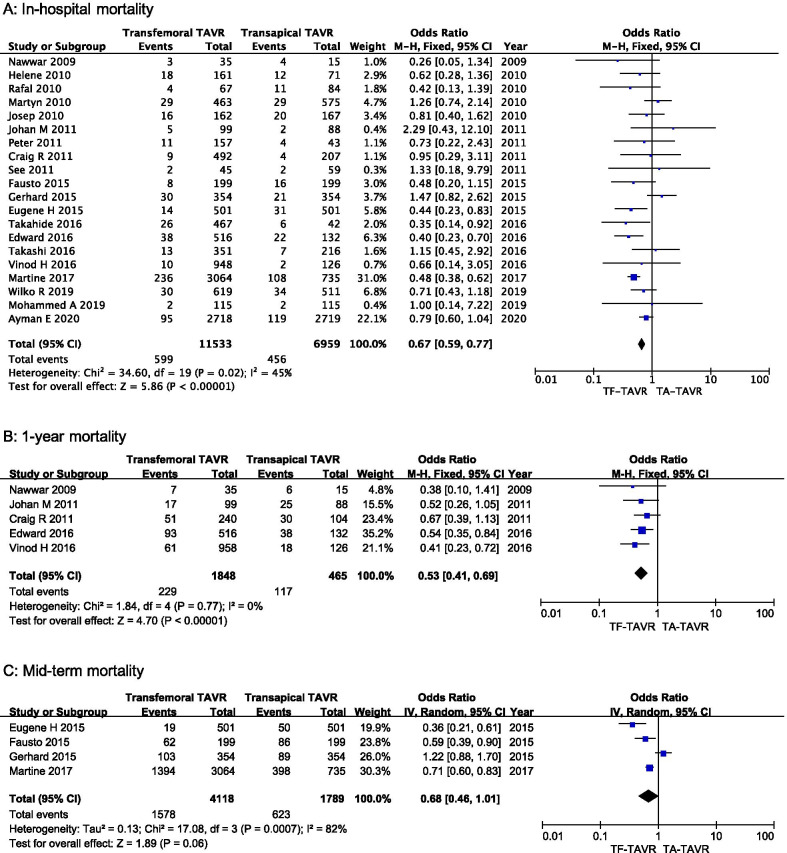
Forest plot of direct comparison meta-analysis of postoperative mortality rate between TF-TAVR versus TA-TAVR: **a** in-hospital mortality evaluated by M-H fixed-effect model; **b** 1-year mortality evaluated by M–H fixed-effect model; **c** mid-term mortality evaluated IV random-effects model. TF: transfemoral; TA: transapical; TAVR: transcatheter aortic valve replacement; OR, odds ratio; CI, confidence interval; H–M, Mantel–Haenszel; IV, inverse variance

Postoperative in-hospital mortality was reported in 20 studies. One RCT and 19 observational studies with 18,492 patients were included. In the pooled analysis, in-hospital mortality was significantly lower with TF-TAVR compared with TA-TAVR (OR = 0.67, 95% CI 0.59–0.77, P < 0.001, Fig. 2a).Postoperative 1-year mortality was reported in 5 studies. One RCT and 4 observational studies with 2,313 patients were included. In the pooled analysis, 1-year mortality remained significantly lower in TF-TAVR compared with TA-TAVR (OR = 0.53, 95% CI 0.41–0.69, P < 0.001, Fig. 2b). Postoperative mid-term mortality was reported in 4 observational studies with 5,907 patients. The pooled analysis did not demonstrate a statistically significant difference in the risk of mid-term mortality when comparing TF-TAVR versus TA-TAVR (OR = 0.68, 95% CI 0.46–1.01, P = 0.06, Fig. 2c).

### Morbidity and other complications

**Fig. 3:**
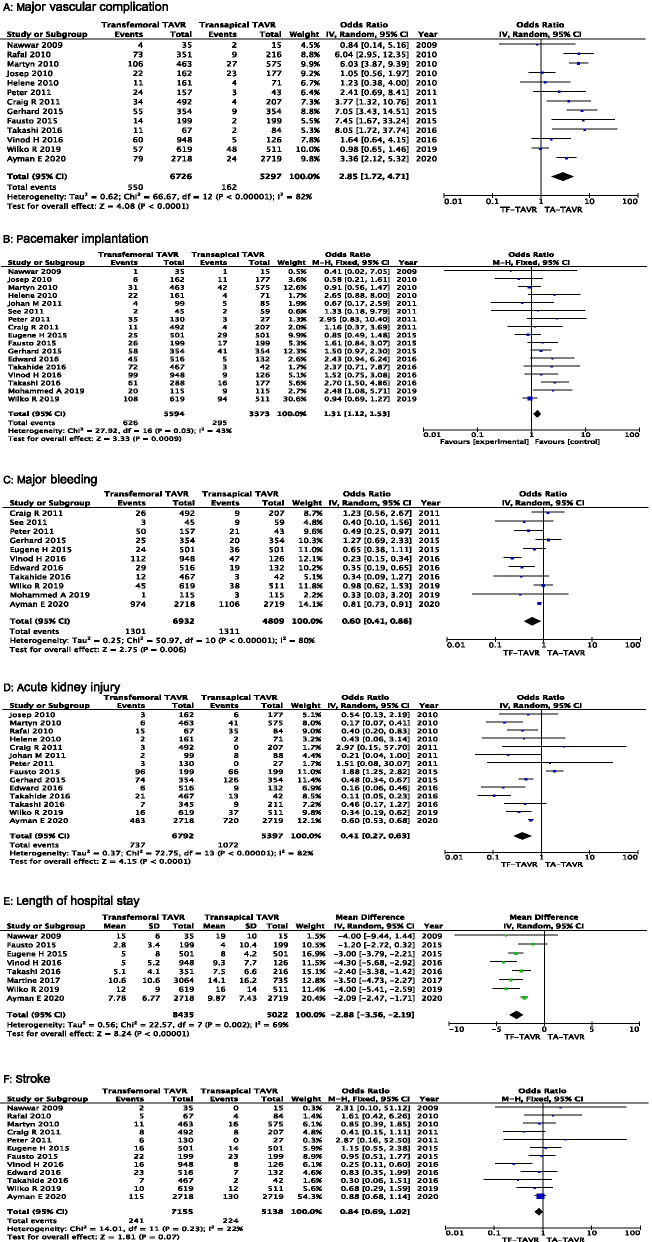
Forest plot of direct comparison meta-analysis of postoperative event rate between TF-TAVR versus TA-TAVR: **a** major vascular complication evaluated by IV random-effect model; **b** pacemaker implantation evaluated by M–H fixed-effect model; **c** major bleeding by IV random-effect model. TF: transfemoral; TA: transapical; TAVR: transcatheter aortic valve. **d** acute kidney injury evaluated by IV random-effect model; **e** length of hospital stay evaluated by IV random-effect model; **f** stroke evaluated by M–H fixed-effect model. TF: transfemoral; TA: transapical; TAVR: transcatheter aortic valve replacement; OR, odds ratio; CI, confidence interval; H–M, Mantel–Haenszel; IV, inverse variance

Results for the other outcomes are summarized in Fig. 3. The pooled analysis of 13 studies (12,023 patients) demonstrated a higher risk of major vascular complication with TF-TAVR compared with TA-TAVR (OR = 2.85, 95% CI 1.72–4.71, P < 0.001, Fig. 3a). Meanwhile, in the pooled analysis of 17 studies (n = 8,967), there was a significantly higher incidence of pacemaker implantation in the TF-TAVR group (OR = 1.31, 95% CI 1.12–1.53, P < 0.001, Fig. 3b).

On the other hand, pooled analyses revealed that TF-TAVR was associated with lower risk for major bleeding (11 studies, 11,741patients, OR = 0.60, 95% CI 0.41–0.86, P = 0.006, Fig. 3c) and acute kidney injury (14 studies, 12,189 patients, OR = 0.41, 95% CI 0.27–0.63, P < 0.001, Fig. 3d), as well as shorter length of hospital stay (8 studies, 13,457 patients, mean difference = -2.88 days, 95% CI -3.56 to -2.19, P < 0.001, Fig. 3e). Pooled analysis of 12 studies (12,293 patients) demonstrated no statistically significant difference in the risk of stroke among patients assigned to TF-TAVR versus TA-TAVR (OR = 0.84 95% CI 0.69–1.02, P = 0.07, Fig. 3f).

Funnel plots for each outcome are shown in Additional file 1: Fig. S1. No significant publication biases were detected. The results of the sensitivity analyses were consistent with the primary analysis for all the endpoints (Additional file 1: Table S1).

## Discussion

Since its first clinical application in 2002, TAVR has gone through several generations of evolution and expanded rapidly to be a nonnegligible alternative to SAVR in patients with high and intermediate procedural risk. It is foreseen that the number of TAVR procedures will continue to increase with the appearance of novel generations of prosthetic valves and delivery devices, as well as expanded indications from high-risk and inoperable elder patients to younger and low-risk patients [35]. In addition, patients with native aortic valve regurgitation can also be treated successfully with TAVR with randomized trials under designing aimed to prove its mid and long-term performance [36, 37]. Minimally invasive surgery is the most attractive merit of TAVR, which makes TF approach the preferred one, given its less inherent risk for postoperation complications by avoiding more invasive steps such as mini-thoracotomy and left ventricular puncture in TA-TAVR. However, despite the improvement in device profiles and procedure techniques, TF access is faced with technical limitations such as the sheath size and the prosthetic orifice area, which cannot be performed in a considerable proportion of patients. Thus, TA access remained applicable during these scenario in clinic practice. The attendant problem is whether these two different approaches have similar performances. Several previous studies have compared the outcomes of TF-TAVR versus those of TA-TAVR based on observational studies with relatively early data (before 2014) and small sample size and drew contradictory results. Panchal et al. reported that 1-year mortality was similar in both approaches while TF approach resulted in lower 30-day mortality [38]. Liu et al. concluded a comparable result for both 30-day and 1-year mortality [39]. Conversely, Ghatak et al. reported superior 30-day and mid-term mortality with TF-TAVR [40]. The discrepancy will cause dilemma and confusion for treatment decisions.

By pooling data from 1 RCT and 18 observational trials, this large sample volume meta-analysis has included the latest and most comprehensive studies in this area. The results demonstrated that the mid-term deaths and stroke incidences were comparable between TF-versus TA-TAVR, while the number of early deaths (30-day and 1-year) was smaller with TF approach than with TA approach. Since there was no obvious difference in patient risk factors (using STS or EuroSCORE in different studies) between two approaches, it may be speculated that the higher early mortality with TA approach could be related to (i) the physical damage to the myocardium through direct puncture of the apex, (ii) surgical chest trauma, and (iii) effects of general anesthesia. TA-TAVR has been also associated with cardiac biomarkers level elevation and poorer cardiac function improvement [41]. These perioperative complications appeared to have early rather than mid to long-term consequences. Therefore, performance of patients in TA group surviving beyond the early postoperative convalescence would gradually catch up with those in TF group.

The postoperative complications requiring special attention during the early convalescence in patients with TA-TAVR are acute kidney injury and major bleeding events since they present significantly higher occurrences than those in TF group. Worth noting, these two complications had previously been identified as predictors of adverse outcome including mortality and longer hospital stay following TAVR [42, 43]. Postoperative renal dysfunction is not uncommon in TAVR patients due to the side-effect of contrast media and inadequate renal perfusion during the hemodynamic alterations during the procedure. Moreover, the high incidence of AKI in TA-TAVR patients can also be ascribed to the transfusion for there were more major bleeding events in TA group. Transfusion has been proved to be an independent predictor of AKI as it is associated with the coadministration of some other causative molecular and cellular substances causing kidney injury, such as interleukin-8 which typically accumulates in stored packed red cells [44]. So it is reasonable to emphasize the importance of close monitoring of perioperative renal function, as well as a strict surgical discipline in the execution of TA-TAVR by—among others—strict control of hemostasis, especially the puncture site on heart.

Despite accumulated experiences and meticulous efforts to redesign the transcatheter prosthesis and sheath (smallest sheath size has been reduced to 14 Fr or equivalent nowadays), vascular complications and conduction abnormalities were increasingly observed with TF-TAVR. Consistently, higher incidence of vascular complications may reflect the inherent defect of TF approach. Recent echo-guided puncture and closure devices had emerged to ensure proper entry and hemostasis of the femoral artery. However, vascular complications may be inevitable in patients with poor arterial condition and the key for prevention is a comprehensive preoperative assessment and proper patient selection. On the other hand, the higher incidence of pacemaker implantation in TF-TAVR patients may lead to adverse clinical sequelae on their long-term outcomes through the loss of atrioventricular synchrony, lack of physiological rate control, and unphysiological right ventricular stimulation. The mechanism of conduction tissue injury is speculated to be due to the mechanical pressure from metal struts. Some researchers suggested that the likelihood of pacemaker implantation differs according to valve design (significantly higher with self-expandable valves, marginally elevated with balloon-expandable valves) [45, 46]. The higher rate of pacemaker implantation in TF-TAVR patients may be associated with the position difficulty and repeated attempts during the angiographic deployment. Hence, further technical refinements in valve and sheath design as well as precise image-guided puncture and positioning are warranted to improve the performance of TF-TAVR given the significant impact of conduction abnormalities and major vascular complications.

Several limitations to the current meta-analysis need to be acknowledged. The baseline characteristics between the two approaches could not be compared entirely, attributed to the inherent nature of the meta-analysis. The use of various type and generations of prostheses in these studies may limit the validity of the findings in the current meta-analysis, since there are certain, albeit minor, differences in different TAVR prostheses. Part of these trials were small volumes with limited data to assess outcomes, thus some of these studies may have been underpowered. The overall follow-up period was short to intermediate, that is why some other crucial outcomes such as durability of the prostheses is not investigated. Because of the unavailability of combined MACCE outcomes data in the original studies, we were unable to include them in our analysis. Finally, the data analyzed in this study are mainly observational and with only one randomized concerning transfemoral and transapical access, leading to an indication bias. However, in the shortage of randomized data, the findings of our analysis can further advise the practice of TAVR clinicians and influence future studies. In the future, more randomized controlled trials and comprehensive registries with longer follow-up (> 5-year) will help us to better define the safety and durability, and subsequently, indications of the technique, and the respective places of transfemoral and transapical approaches.

## Conclusions

Nowadays, not only elder patients at very high surgical risk or with contraindications to SAVR, but also younger and low-risk patients with aortic valve disease will benefit from TAVR, The availability of both transfemoral and transapical approaches can increase the number of patients who can be treated. In our analysis, the mid-term mortality and risk of stroke are similar with TA- and TF-TAVR. TF-TAVR has significantly less early mortality, but with a higher incidence of major vascular complications and pacemaker implantation. On the other hand, TA-TAVR is associated with a significant increase in the risk of major bleeding, AKI, and has a longer length of hospitalization. Hereby, both TA and TF are effective approaches with satisfactory short to mid-term outcomes for patients need TAVR treatment. However, it is reasonable to make the approach choice based on detailed individualized evaluation and the experience of local heart teams.

## Supplementary Information

**Additional file 1:** **Table S1:** Results of sensitivity analysis

**Additional file 2: Figure S1:** Funnel plot of comparison meta-analysis outcomes between TF-TAVR versus TA-TAVR: (A) Major bleeding events; (B) 30-day mortality; (C) Major vascular complications; (D) Pacemaker implantation; (E) Acute kidney injury; (F) Length of hospital stay; (G)Mid-term mortality; (H) 1-year mortality; (I)Stroke. Guideline for methodology: PRISMA 2020_Checklist

## Data Availability

The datasets generated and/or analysed during the current study are available in the PubMed (www.pubmed.ncbi.nlm.nih.gov), ClinicalKey (www.clinicalkey.com), the Web of Science (www.webofknowledge.com) and Google Scholar (www.scholar.google.com).

## Abbreviations

AKI: Acute kidney injury
CI: Confidence interval
MACCE: Main adverse cardiovascular and cerebrovascular events
OR: Odds ratio
RCT: Randomized controlled trial
SAVR: Surgical aortic valve replacement
STS: The society of thoracic surgeons
TA: Transapical
TAVR: Transcatheter aortic valve replacement
TAVI: Transcatheter aortic valve implantation
TF: Transfemoral
