# Supplementary material for: Dose approach matter? A meta-analysis of outcomes following transfemoral versus transapical transcatheter aortic valve replacement
Source: BMC Cardiovasc Disord. 2021 Jul 28;21:358. doi: 10.1186/s12872-021-02158-4 (PMC8320184; doi:10.1186/s12872-021-02158-4)
Supplement: Supplementary file 1 — Additional file 1: Table S1: Results of sensitivity analysis [file 12872_2021_2158_MOESM1_ESM.pdf]

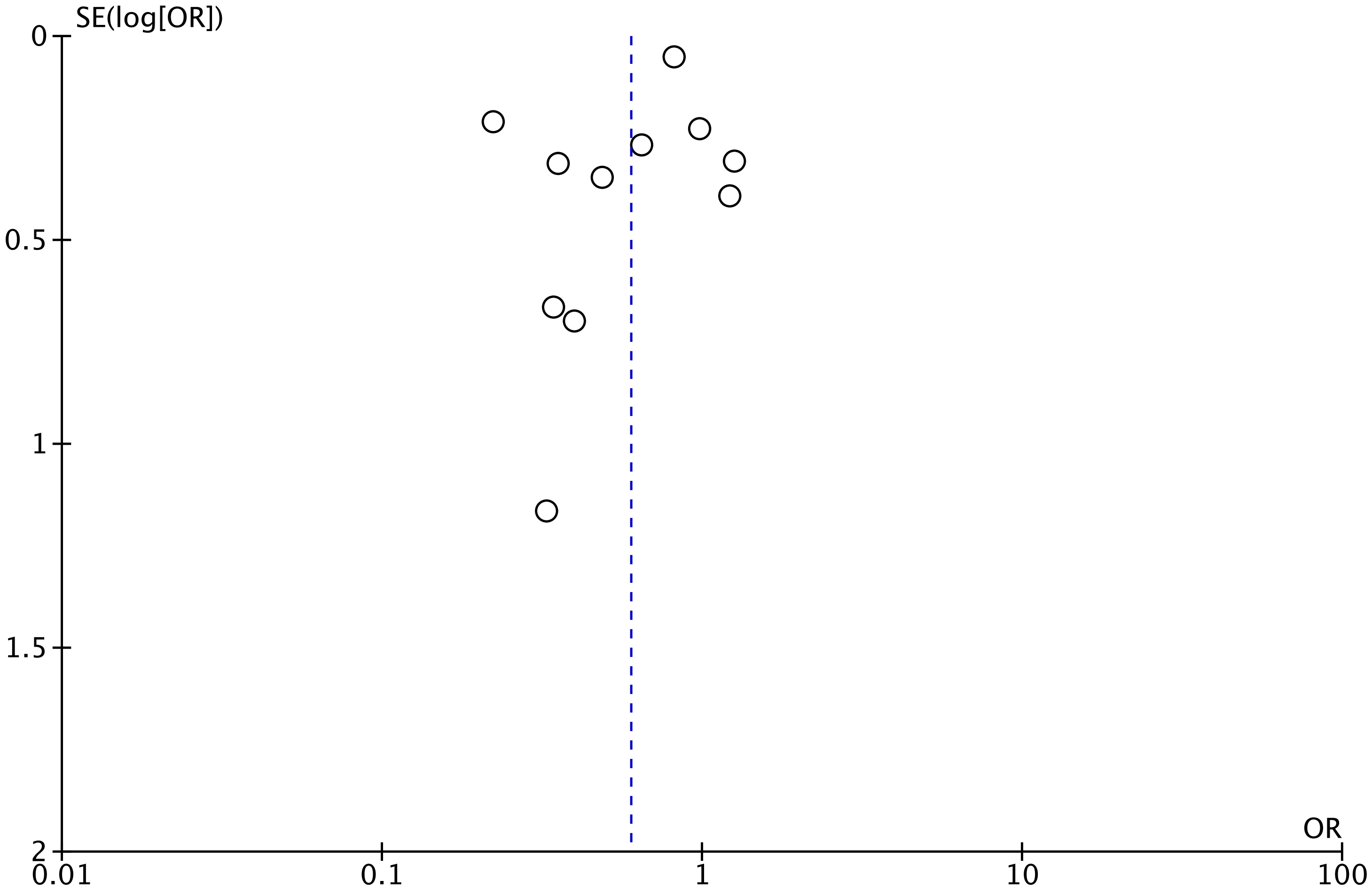


Funnel plot of comparison meta-analysis outcomes between TF-TAVR versus TA-TAVR：(A) Major bleeding events

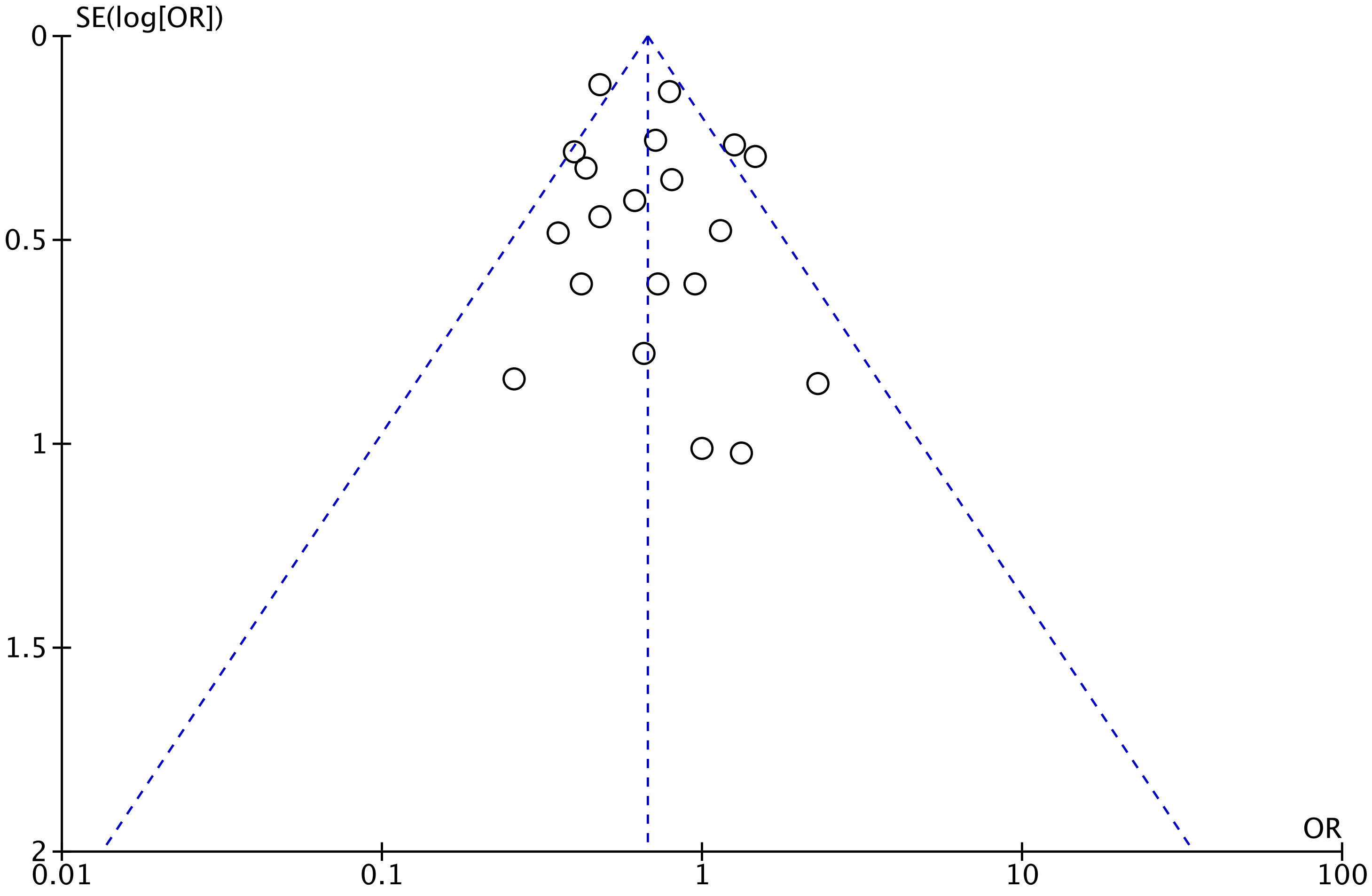


Funnel plot of comparison meta-analysis outcomes between TF-TAVR versus TA-TAVR：(B) 30-day mortality

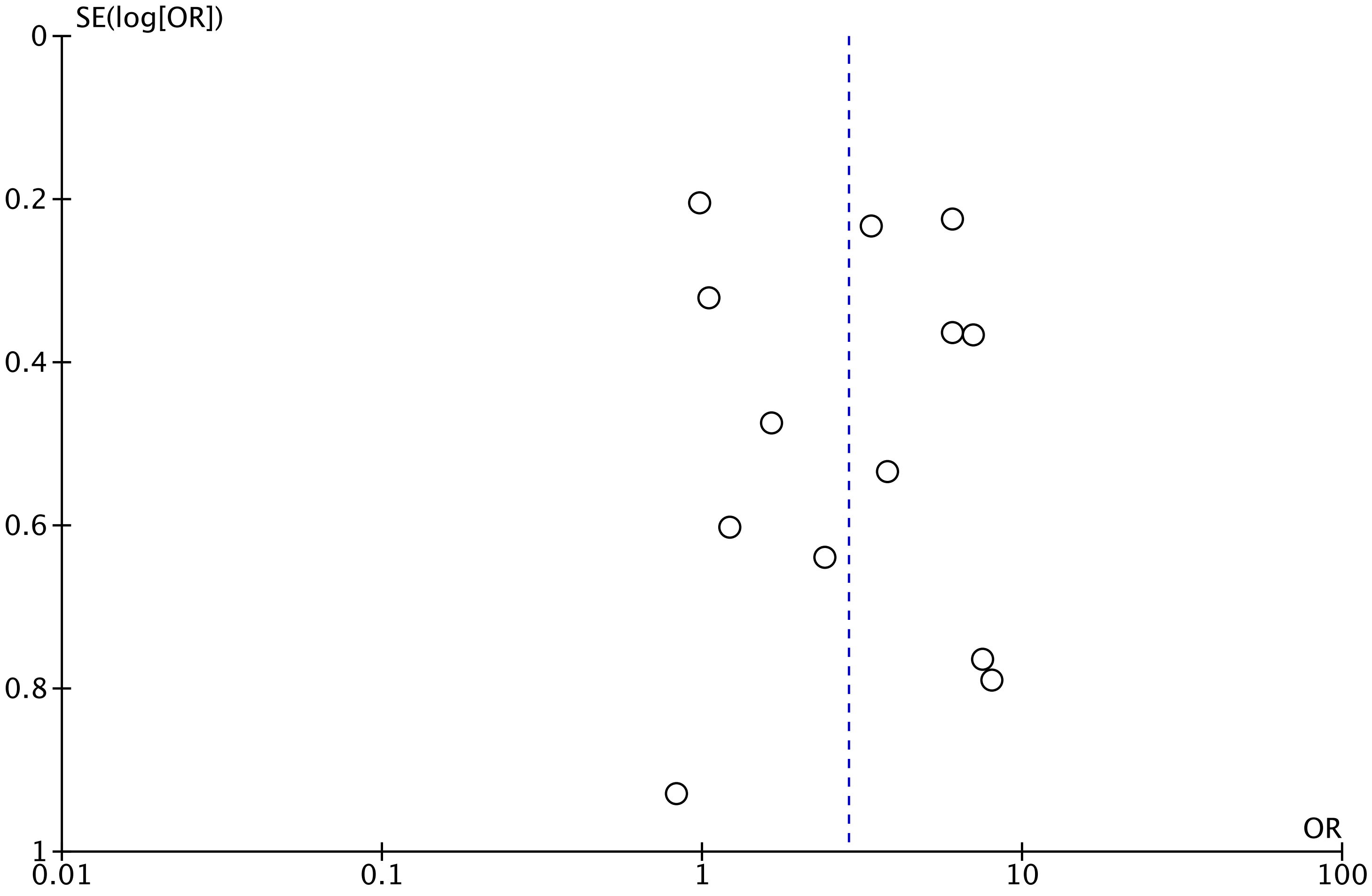


Funnel plot of comparison meta-analysis outcomes between TF-TAVR versus TA-TAVR：(C) Major vascular complications

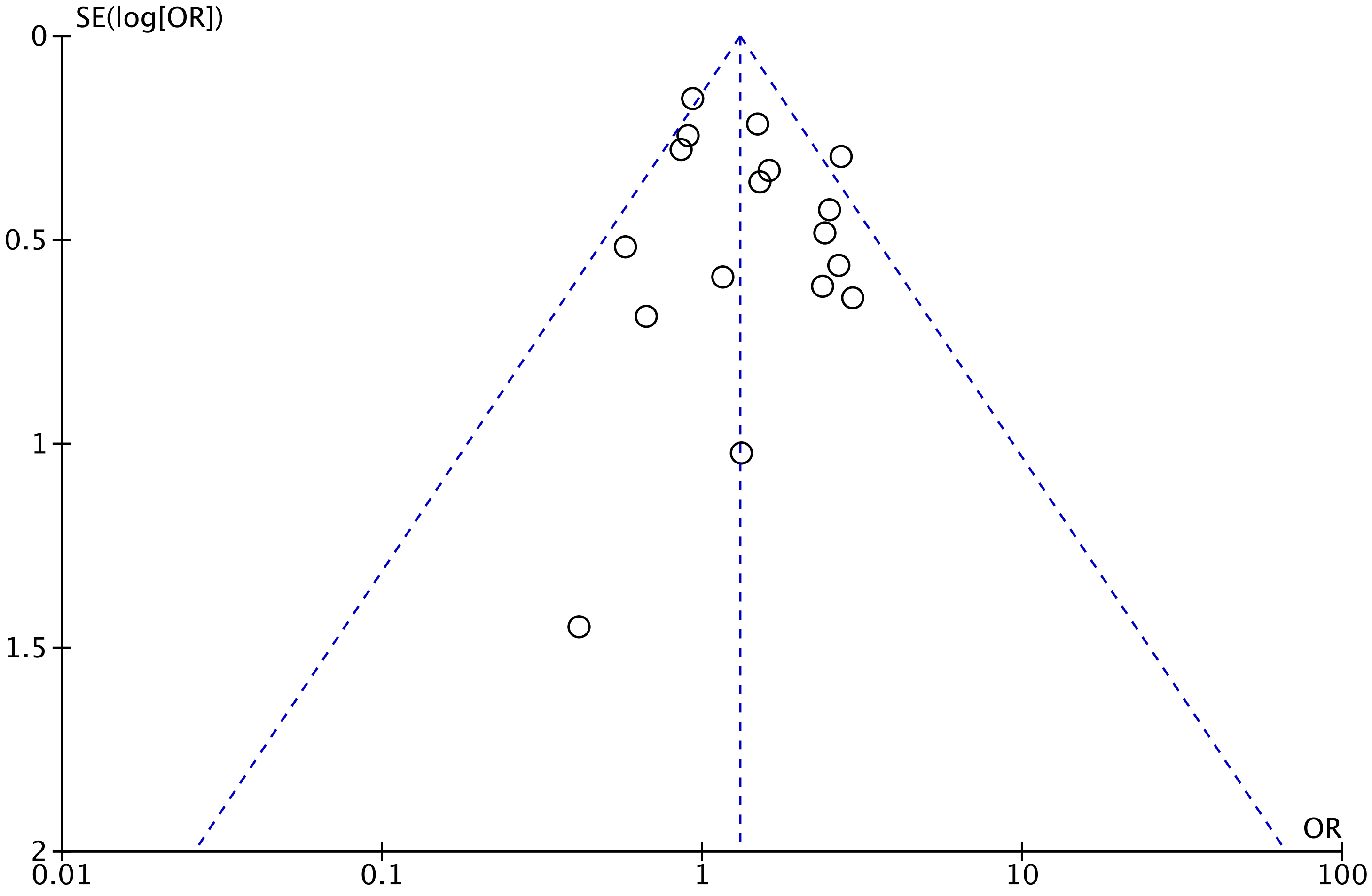


Funnel plot of comparison meta-analysis outcomes between TF-TAVR versus TA-TAVR：(D) Pacemaker implantation

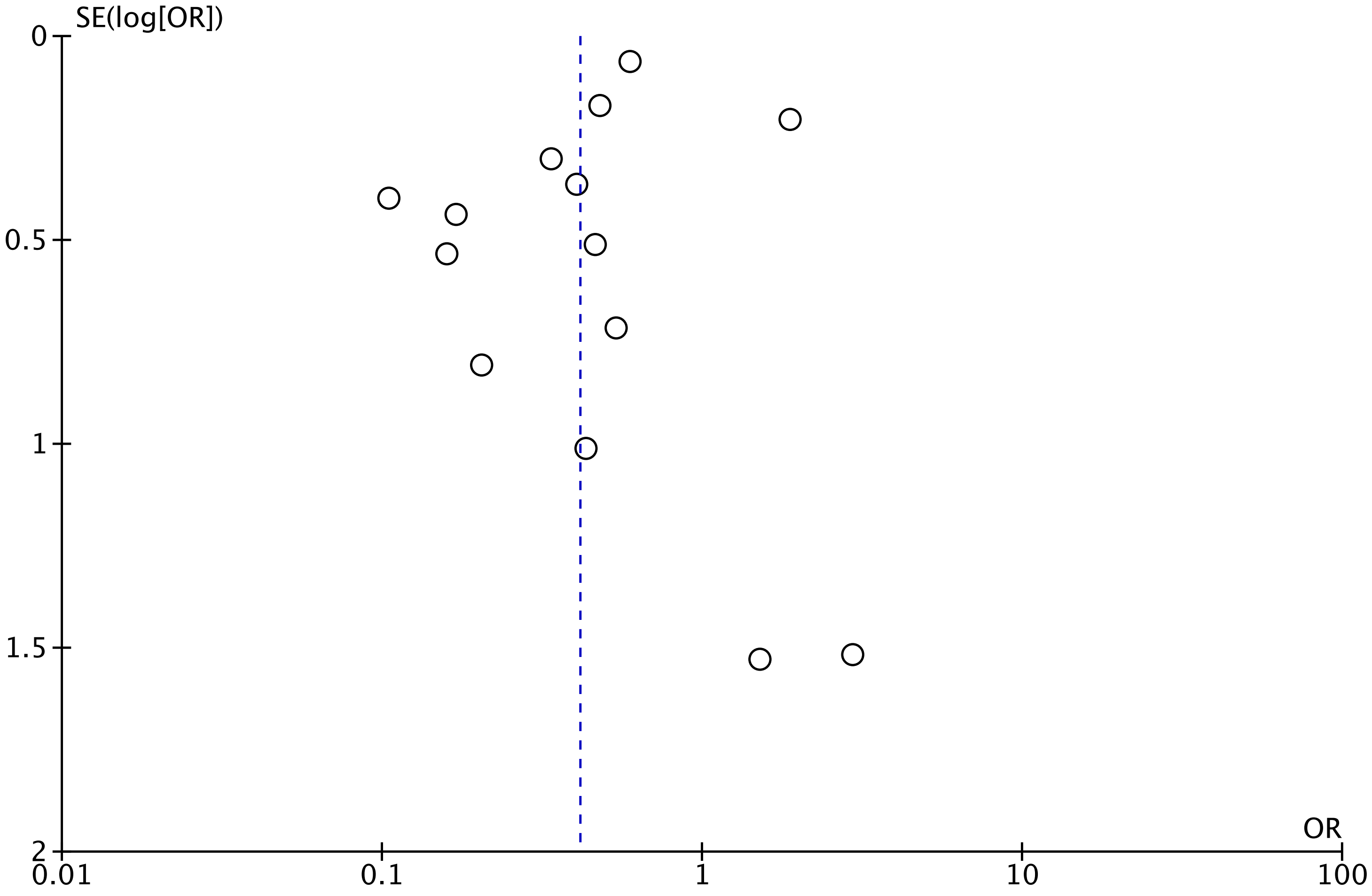


Funnel plot of comparison meta-analysis outcomes between TF-TAVR versus TA-TAVR：(E) Acute kidney injury

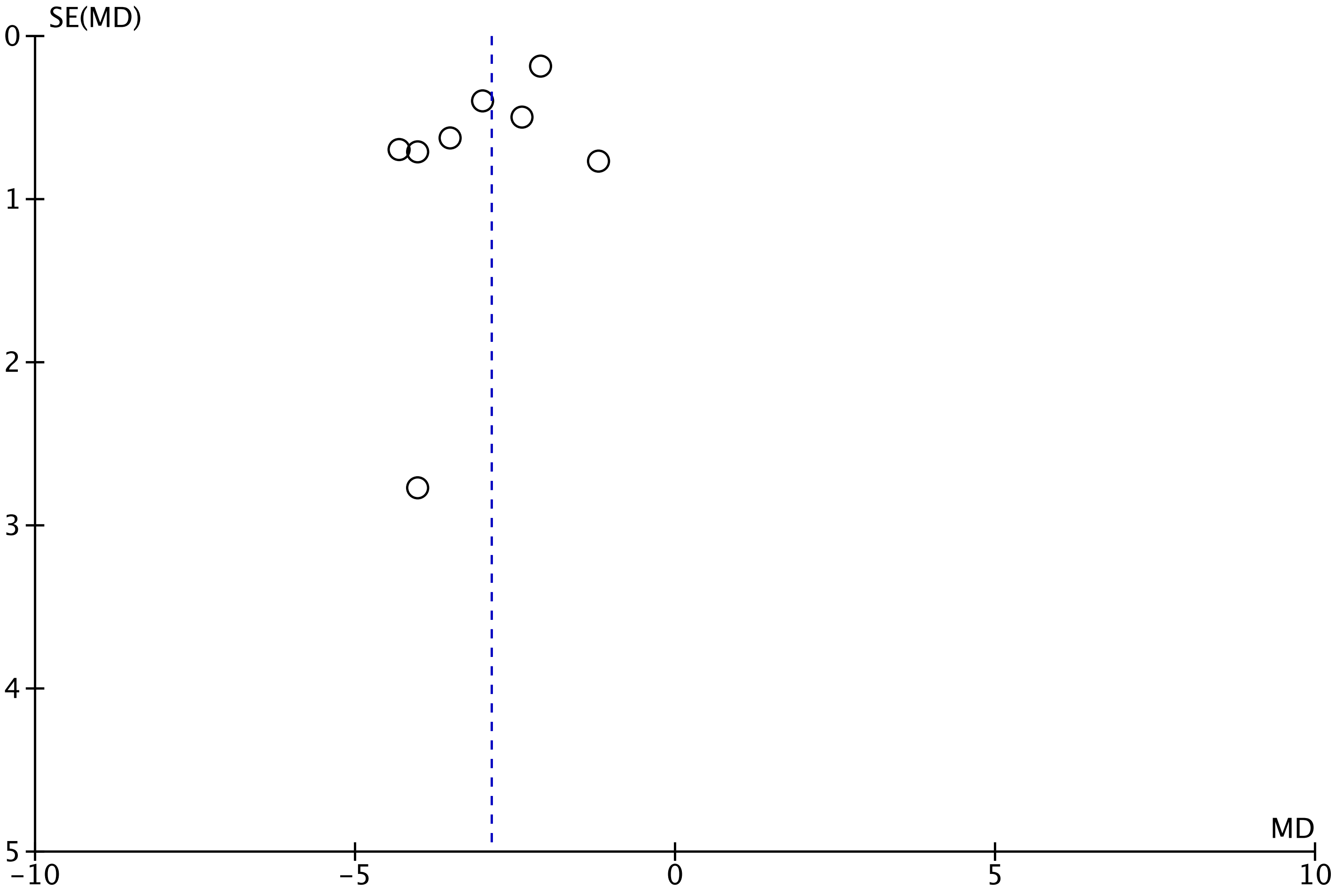


Funnel plot of comparison meta-analysis outcomes between TF-TAVR versus TA-TAVR：(F) Length of hospital stay

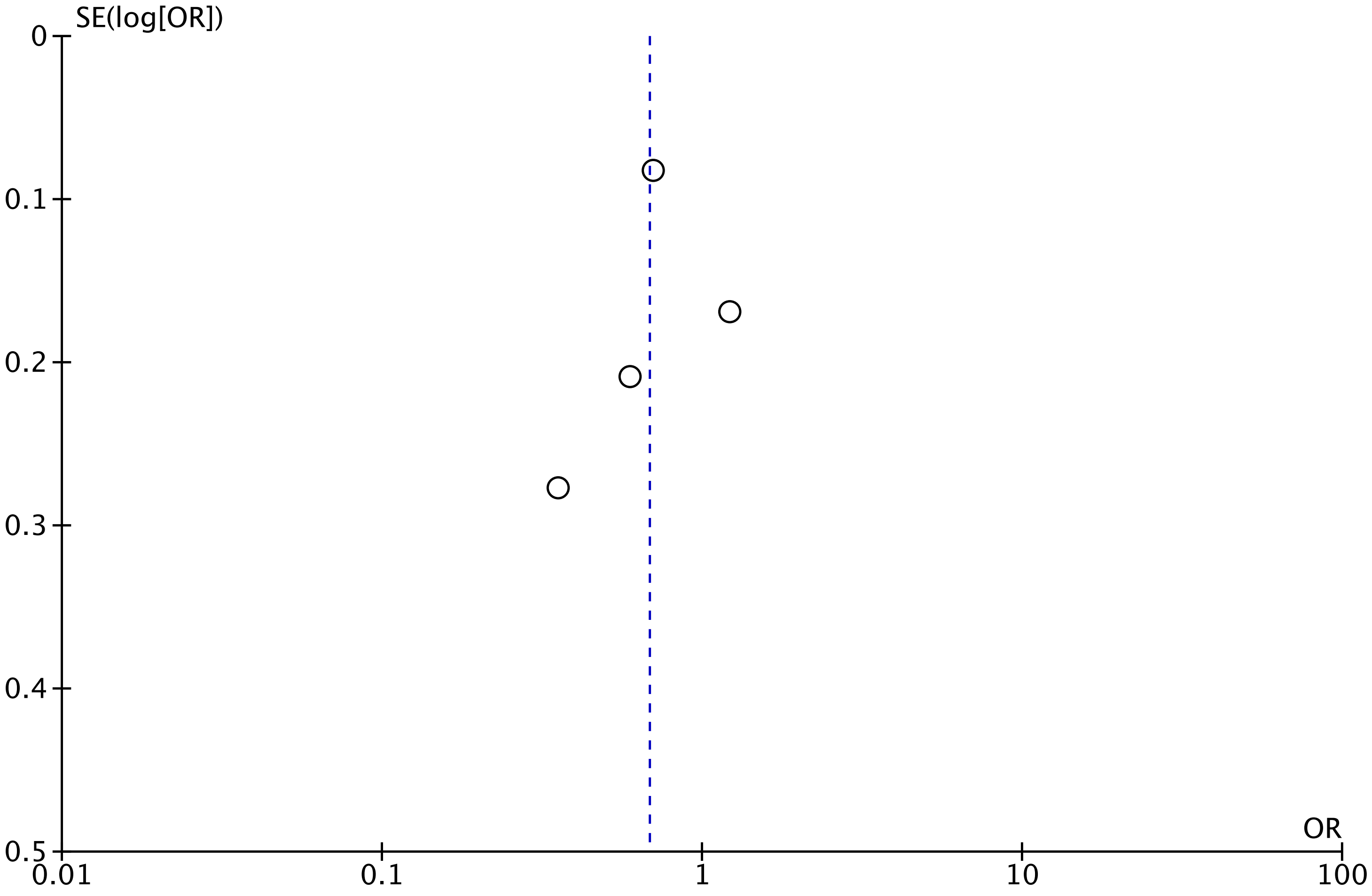


Funnel plot of comparison meta-analysis outcomes between TF-TAVR versus TA-TAVR：(G)Mid-term mortality

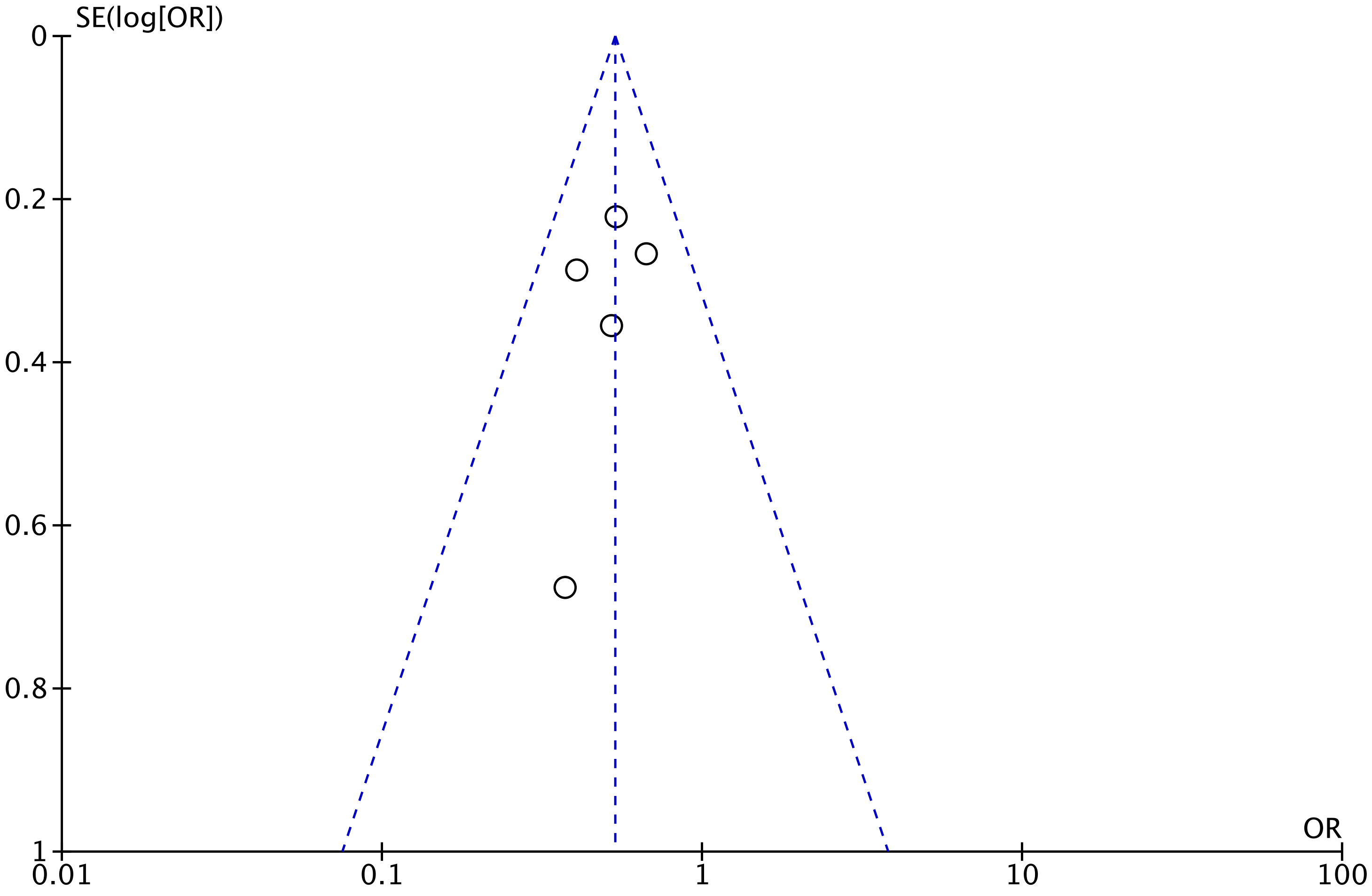


Funnel plot of comparison meta-analysis outcomes between TF-TAVR versus TA-TAVR：(H) 1-year mortality

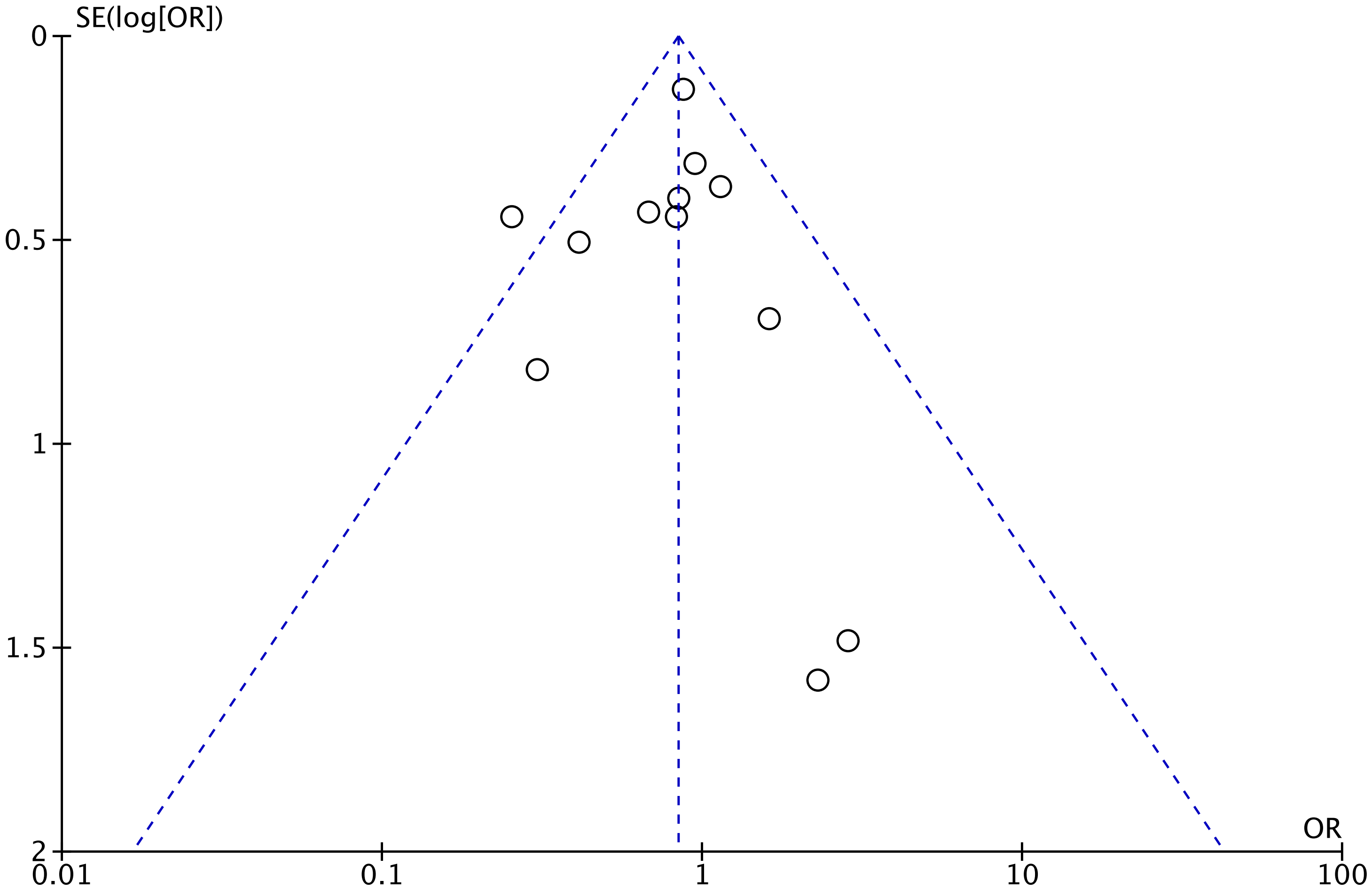


Funnel plot of comparison meta-analysis outcomes between TF-TAVR versus TA-TAVR：(I)Stroke
